# A novel cooperative distributed secondary controller for VSI and PQ inverters of AC microgrids

**DOI:** 10.1016/j.heliyon.2019.e01823

**Published:** 2019-06-27

**Authors:** F. Doost Mohammadi, H. Keshtkar, A. Dehghan Banadaki, A. Feliachi

**Affiliations:** a Christopher Newport University, Newport News, VA, 23606, USA; b California Polytechnic State University, San Luis Obispo, CA, 93407, USA; c West Virginia University, Morgantown, WV, 26505, USA

**Keywords:** Electrical engineering, Microgrid control, Microgrids, Cooperative control, Sensitivity analysis, Zone dedications, PQ and VSI inverters

## Abstract

This paper proposes a novel cooperative secondary control strategy for microgrids which is fully distributed. There is a two-layered coordination, which exists between inverter based DGs of both types, i.e. Voltage Source Inverter (VSI) and Current Source Inverter (CSI), also called PQ inverter. In first layer of the proposed two-layered cooperative control strategy, VSIs will take care of the primary average voltage regulation by implementing the average consensus algorithm (ACA); then in the second layer of control, the PQ inverters will improve the voltage quality of the microgrid while maintaining the average voltage of buses at the same desired level. Zone dedication algorithm is utilized in the second layer for voltage quality purposes based on sensitivity analysis. The sensitivity analysis is based on Simplified Jacobian matrix and the result of that is used to define the zone related to each DG in the microgrid. The goal of this zone dedication is to assign loads to the DGs that can compensate their changes with less effort (generating less power) than the others. There are two major contributions in this paper; 1- PQ inverters are effectively involved to increase microgrids capacity for better power management by introducing sensitivity to the PQ inverters set-point. This is defined based on the structure of the microgrid and takes into account the location of load changes. 2- The proposed strategy not only focuses on transient response but also improves the steady state response which smooths the voltage profile of the system while keeping the average voltage at the same desired level.

The algorithm has been applied to a 13 bus system with a fully distributed communication in which each VSI inverter only communicates with its immediate neighbors and each PQ inverter is only in touch with associated bordering agents. The conclusive results verify that the proposed control strategy is an effective way to control the microgrid's voltage to have a smoother and stable voltage profile. The analysis also confirms the robustness of the proposed cooperative control in presence of possible time delays.

## 1 Introduction

Microgrids are a smaller scale of the traditional power system, which has been involved with ever-increasing renewable energies during the last years. Faster response, lower power loss, lower carbon dioxide emission and supplying critical loads are counted as the advantages of the microgrids. Renewable energies such as PVs and Wind Turbines are connected to the microgrid with either VSI or PQ inverters; VSI inverters are responsible for regulating the voltage and frequency of the microgrid while PQ inverters provide a specific amount of active and reactive power for the microgrid. These inverters do not have much inertia, subsequently they could bring challenges and complexities to the microgrid's control such as voltage instability.

As mentioned, VSI inverters are mainly responsible for voltage and frequency regulations so in order to achieve a proper control algorithm especially in an islanded mode, primary and secondary control has been used in the literature [1] for VSIs. In the primary control loop of VSI, the voltage and frequency of the system will be adjusted based on the droop control settings to maintain system's stability. In traditional power system, proportional load sharing among synchronous machines will be done based on their ratings without any communication link being required. This behavior has been simulated by DGs. In fact, Droop technique mimics the synchronous machine behavior in case of having any load change [1, 2, 3].

However, the primary control is not enough to bring back the system to its nominal values. Therefore, in the secondary control loop of VSIs, the voltage and frequency set-point will be adjusted to setback the voltage and frequency of the system to their nominal values [4, 5]. In literatures, the primary control is usually the same while for the secondary loop, the control methods are different consisting of centralized [6], decentralized [7] or a distributed one [8, 9]. The distributed controller has been used here since unlike the centralized one, it is neither computationally expensive nor unreliable in case of one point of failure [10]. Additionally, most papers are focused either on controlling the VSIs [4, 5] or PQ inverters [7]. Even though there are few papers considering both, but the PQ inverter is not dynamically involved, for example in [1], a cooperative secondary controller has been proposed for VSIs and PQs however the set-points for all PQ inverters are fixed values defined by the operator assuming the same ratio for all of them. In other words, they produce a constant power based on their ratings no matter what is the structure of the microgrid and where the load change has happened. The sensitivity analysis for considering the structure of the microgrid is investigated in this paper for control purposes. In [11] and [12], Jacobian based methods for sensitivity analysis have been proposed for transmission system. In [13], another approach for sensitivity analysis has been done in distribution system but the concept is based on the conventional assumption for power systems that one source can be assumed to be a slack bus to supply all remaining power loss of the system. The method that is introduced and formulated here does not need to evaluate the Jacobian matrix iteratively and it does not assume any single bus as a slack one either, which is more reasonable for microgrids that the sources are of smaller size than traditional systems.

The last part is evaluating the robustness of the control method. In literatures, the control strategies of the inverter-based microgrids are tested against different uncertainties related to the control signals [14, 15, 16]. In this paper, the most important parameters that affects the robustness of the control system i.e. stability against delays in communication signals, and plug-and-play capability are discussed.

The salient features and main contributions of the proposed cooperative controller are as follows:

➢ The proposed cooperative secondary control is fully distributed such that each DG only talks to its immediate neighbors.
➢ In the proposed control, the layer related to PQ inverters effectively involves them to increase microgrids capacity for better power management. It is no longer a static layer but it is dynamically cooperating with the VSI inverters to balance loads and supply. In other words, PQ controller set-points are not manually set by an operator or an expert who utilizes the power ratio without considering the optimal performance. By proposed controller, they are adjusted automatically according to microgrid's condition.
➢ In this paper, sensitivity analysis is formulated in a way that it is not dependent on the operating point of the system but it is calculated based on the topology of the system.
➢ Zone dedication for PQ inverters is introduced based on the sensitivity analysis which is defined according to the structure of the microgrid and takes into account the location of load's change. This is an improvement compared to other similar literatures which focus only on the VSI inverter's control in the secondary control design or consider a very static contribution from PQ inverters without any interaction with microgrid.
➢ The proposed cooperative control between VSI and PQ inverters not only focuses on transient response but also improves the steady state response by smoothing the voltage profile of the system while keeping the average voltage at the same desired level.
➢ The proposed secondary controller is robust in presence of possible time delays in VSI or PQ layer. It is also robust in terms of any failure in its communication devices for any or all of the PQ inverters. In such a case, other VSIs and those PQ inverters with working communication links will take care of the system.

The rest of this paper is organized as follows. At first, problem statement and modeling of inverter-based microgrids are briefly discussed in section 2. In section 3, zone dedication method is discussed and then it is followed by sensitivity analysis in section 4. In section 5, the simulation results are shown for four different cases to verify the effectiveness and robustness of the algorithm. Finally, section 6 concludes the paper.

## 2 Model

### 2.1 Problem statement and modeling of inverter-based microgrids

#### 2.1.1 Problem statement

**Fig. 1:**
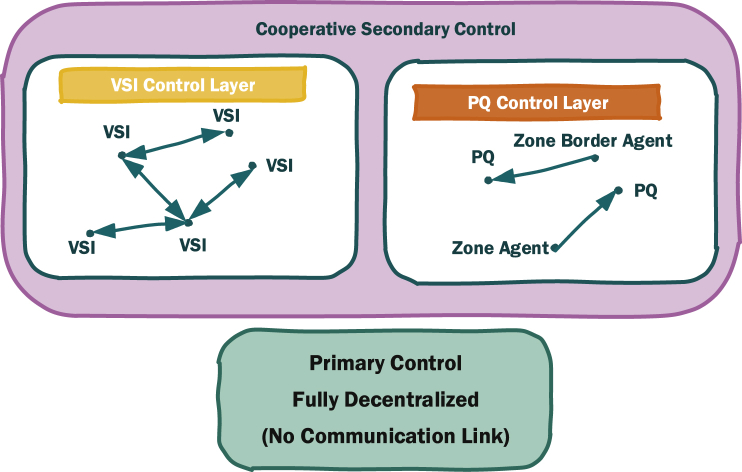
Control layers of the proposed control algorithm.

In this paper, the design of a distributed cooperative secondary control algorithm for microgrids is discussed. As shown in Fig. 1, The control scheme has two levels called primary and secondary where the latter one itself is formed by cooperative operation of two distinct layers of control associated to VSI and PQ inverters.

The conventional method is used as the primary control of the microgrid which is fully decentralized without any communication requirements. It works based on droop characteristics and its automatic and decentralized operation, enables the microgrid to maintain the stability quickly and automatically without relying on communication infrastructure. However, the remaining errors need to be regulated where secondary control comes into play.

As mentioned earlier, the focus of this paper is on secondary control which deals with removing the steady state errors. The first (also the main) layer of secondary control works based on adjusting the nominal set-point of VSI inverters using ACA. Then the second layer involving PQ inverters is added which utilizes sensitivity analysis to improve the performance and steady state response by smoothing the overall voltage profile and keeping all buses voltages closer to the nominal value. These two layers of secondary control work cooperatively and play a significant role in improving the steady state response of the system.

The proposed control method is also flexible in case of having a new DG being added or disconnected from the microgrid. *In PQ control layer*, the PQs should communicate with their border agents to be aware of the amount of power that is flowing in and out of the zone. So if a new PQ inverter is plugged in to the microgrid, it should start communicating with its associated border agent based on the assigned zone. In case of play out, the zone associated with the PQ inverter will be handled by VSI inverters automatically.

*In VSI control layer*, the VSI inverters need to communicate with a few neighboring VSI, not all of them. The connections between neighbors are defined by the network design problem to achieve a trade-off between the communication cost and the agreement time delay for consensus algorithm. However, since average consensus algorithm is being used it makes the plug in and play out operation very easy since a new DG just needs to get in touch with at least one of the available VSIs in the microgrid to be aware of the agreement values.

It should be noticed that the primary control is robust and fully decentralized without any communication for primary coordination of VSIs. However, communication requirement for secondary control layers necessitates the delay response analysis due to the existence of possible time delays.

#### 2.1.2 Modeling of inverter-based microgrids

Controlling the inverters in a microgrid is the critical part of the control methodology and having a proper modeling is the prerequisite for the further analysis. Inverter-based DGs can work either in VSI mode or in PQ mode which their modeling is discussed here briefly. More details about modeling section can be found in [17] and [18].

##### 2.1.2.1 Modeling of voltage source inverters (VSI)

**Fig. 2:**
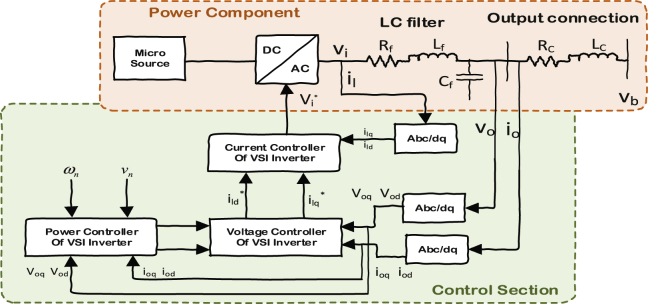
Block diagram of VSI inverter.

The block diagram of a VSI inverter which is shown in Fig. 2, is consisted of power electronic and controlling parts. The LC filter and the output connection for the prior one are formulated in d-q axis in (Eqs. 1, 2 and 3) respectively.

(1) $$\frac{d}{dt}\left[\begin{array}{c} i_{ld}\\ i_{lq} \end{array}\right]=\frac{-R_{f}}{L_{f}}\left[\begin{array}{c} i_{ld}\\ i_{lq} \end{array}\right]i_{ld}+\omega \;\left[\begin{array}{c} i_{lq}\\ -i_{ld} \end{array}\right]+\frac{1}{L_{f}}\left[\begin{array}{c} v_{id}-v_{od}\\ v_{iq}-v_{oq} \end{array}\right]$$

(2) $$\frac{d}{dt}\left[\begin{array}{c} v_{od}\\ v_{oq} \end{array}\right]=\omega \;\left[\begin{array}{c} v_{oq}\\ -v_{od} \end{array}\right]+\frac{1}{C_{f}}\left[\begin{array}{c} i_{ld}\\ i_{lq} \end{array}\right]-\frac{1}{C_{f}}\left[\begin{array}{c} i_{od}\\ i_{oq} \end{array}\right]$$

(3) $$\frac{d}{dt}\left[\begin{array}{c} i_{od}\\ i_{oq} \end{array}\right]=\frac{-R_{c}}{L_{c}}\left[\begin{array}{c} i_{od}\\ i_{oq} \end{array}\right]+\omega \;\left[\begin{array}{c} i_{oq}\\ -i_{od} \end{array}\right]+\frac{1}{L_{c}}\left[\begin{array}{c} v_{od}\\ v_{oq} \end{array}\right]-\frac{1}{L_{c}}\left[\begin{array}{c} v_{bd}\\ v_{bq} \end{array}\right]$$

**Fig. 3:**
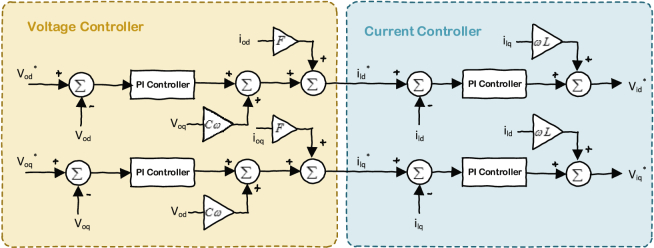
Current and voltage controller of VSI inverters.

Fig. 3 is showing the modeling of the voltage and current controllers which are formulated respectively in (Eqs. 4, 5, 6 and 7). The current controller generates the reference voltage for the inverter and the voltage controller generates the reference point for the current controller. These two controllers will regulate the voltage of the inverter at its own bus to the nominal value by using a PI integrator.

(4) $$\left[\begin{array}{c} {\dot{\phi }}_{d}\\ {\dot{\phi }}_{q} \end{array}\right]=\left[\begin{array}{c} {v^{*}}_{od}\\ {v^{*}}_{oq} \end{array}\right]-\left[\begin{array}{c} v_{od}\\ v_{oq} \end{array}\right]$$

(5) $$\left[\begin{array}{c} {i^{*}}_{ld}\\ {i^{*}}_{lq} \end{array}\right]=F\left[\begin{array}{c} i_{od}\\ i_{oq} \end{array}\right]+\omega_{n}C_{f}\left[\begin{array}{c} -v_{oq}\\ v_{od} \end{array}\right]+K_{pv}\left[\begin{array}{c} {v^{*}}_{od}-v_{od}\\ {v^{*}}_{oq}-v_{oq} \end{array}\right]+K_{iv}\left[\begin{array}{c} \phi_{d}\\ \phi_{q} \end{array}\right]$$

(6) $$\left[\begin{array}{c} {\dot{\mu }}_{d}\\ {\dot{\mu }}_{q} \end{array}\right]=\left[\begin{array}{c} {i^{*}}_{ld}\\ {i^{*}}_{lq} \end{array}\right]-\left[\begin{array}{c} i_{ld}\\ i_{lq} \end{array}\right]$$

(7) $$\left[\begin{array}{c} {v^{*}}_{id}\\ {v^{*}}_{iq} \end{array}\right]=\omega_{n}L_{f}\left[\begin{array}{c} -i_{lq}\\ i_{ld} \end{array}\right]+K_{pc}\left[\begin{array}{c} {i^{*}}_{ld}-i_{ld}\\ {i^{*}}_{lq}-i_{lq} \end{array}\right]+K_{ic}\left[\begin{array}{c} \mu_{d}\\ \mu_{q} \end{array}\right]$$

Power controller in inverter based DGs has been adapted from the synchronous machines in which the increased demand is automatically shared among the machines based on their ratings by an automatic reduction in the rotor speed until the extra power is compensated from the mechanic's parts [2]. In DGs, this can be implemented by changing each VSI's frequency (i.e. phase angle) and voltage with respect to its active and reactive power ratings in (Eq. 8) and (Eq. 9) respectively [2].

(8) $$\omega =\omega_{n}-mP$$

(9) $$v_{o}^{*}=V_{n}-nQ\;\;\;\;\;\;\;\;\;\;\;\;\;\;\;\;\;\;\;$$

These two signals will be given to the inverters to balance the power between the demand and supply in a parallel way without using any communication. However, it is still possible that the voltage or frequency of the system deviates from their optimal set points which will be fixed by using the secondary controller in which $\omega_{n}$and $V_{n}$ are adjusted to bring the voltage and frequency back to their nominal values.

##### 2.1.2.2 Modeling of current source inverters (PQ)

PQs are responsible for regulating the active and reactive power of the microgrid. However, the way that they participate in improving the system has been considered passively. In other words, their participation in producing power is usually set to be a fixed proportionate of their power ratings, for example 20% of their capacity. However, in this paper, the participation of these inverters are actively considered based on the zone that is dedicated to them.

**Fig. 4:**
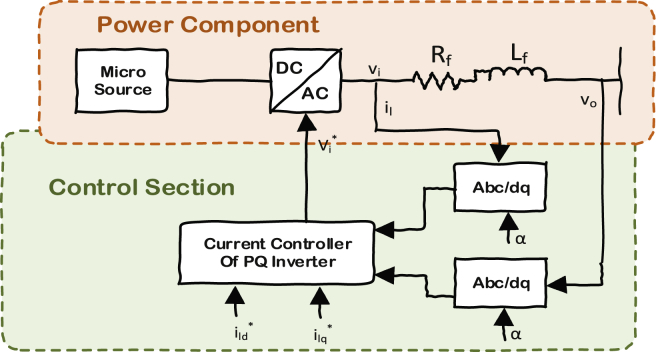
Current controller of PQ inverters.

In Fig. 4, the control block for PQ inverter is shown. PQ controller tries to produce the desired amount of power that has been set as its input. In this controller, the term *α* shown in Fig. 4, will be calculated such that the quadrature term of the output voltage reaches zero (i.e. *v*_*oq*_*=0*). Hence, P and Q of the system will be directly related to the d-q currents in (Eqs. 10 and 11) respectively. This is why PQ controllers are called current controllers too.

(10) $$P=v_{od}i_{ld}+v_{oq}i_{lq}\;\;\;\to As\;\;\;v_{oq}=0\to \;\;\;\;\;\;\;\;\;\;\;P=v_{od}i_{ld}$$

(11) $$Q=v_{oq}i_{ld}-v_{od}i_{lq}\;\;\;\to As\;\;\;v_{oq}=0\to \;\;\;\;\;\;\;\;\;\;\;Q=-v_{od}i_{lq}$$

There is a PI controller inside the current controller block, which regulates the PQ's (i.e. current) references (Eqs. 12 and 13).

(12) $$\left[\begin{array}{c} {\dot{\gamma }}_{d}\\ {\dot{\gamma }}_{q} \end{array}\right]=\left[\begin{array}{c} {i^{*}}_{ld}\\ {i^{*}}_{lq} \end{array}\right]-\left[\begin{array}{c} i_{ld}\\ i_{lq} \end{array}\right]$$

(13) $$\left[\begin{array}{c} {v^{*}}_{id}\\ {v^{*}}_{iq} \end{array}\right]=\left[\begin{array}{c} v_{od}\\ v_{oq} \end{array}\right]+\omega_{n}L_{f}\left[\begin{array}{c} -i_{lq}\\ i_{ld} \end{array}\right]+K_{pc}\left[\begin{array}{c} {i^{*}}_{ld}-i_{ld}\\ {i^{*}}_{lq}-i_{lq} \end{array}\right]+K_{ic}\left[\begin{array}{c} \gamma_{d}\\ \gamma_{q} \end{array}\right]$$

Finally, the equations for the RL filter of the inverters are formulated in (Eq. 14).

(14) $$\frac{d}{dt}\left[\begin{array}{c} i_{ld}\\ i_{lq} \end{array}\right]=\frac{-R_{f}}{L_{f}}\left[\begin{array}{c} i_{ld}\\ i_{lq} \end{array}\right]+\omega_{com}\;\left[\begin{array}{c} i_{lq}\\ -i_{ld} \end{array}\right]+\frac{1}{L_{f}}\left[\begin{array}{c} v_{id}-v_{od}\\ v_{iq}-v_{oq} \end{array}\right]$$

## 3 Methods

### 3.1 Zone dedication to PQ inverters

The consensus-based [19] control that has been introduced in [1], involves VSI inverters but there is no contribution from PQ inverters. In fact, they provided a constant amount of active and reactive power no matter what was happening in the microgrid. However, in this paper, PQ inverters are also involved in improving the voltage profile by dedicating appropriate zones to them. Different zones are defined in a way that each zone includes one of the inverters. After dedicating a zone to an inverter (whether it is PQ or VSI), it is desirable that each inverter takes the responsibility of load changes inside its own zone.

**Fig. 5:**
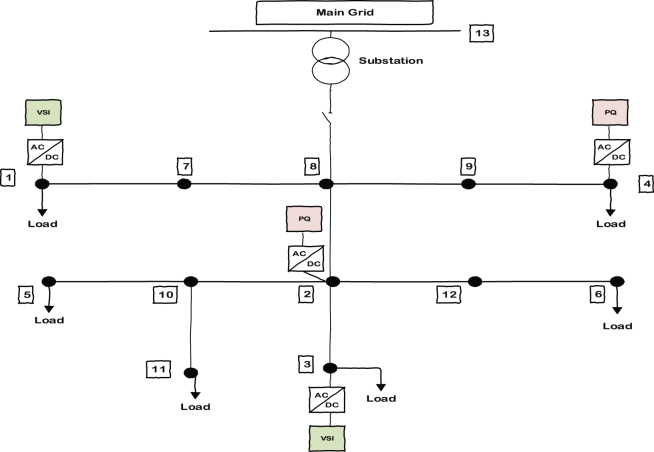
Understudied 13 bus microgrid with two PQs and two VSIs.

Fig. 5 shows the understudied 13 bus microgrid consisting of four inverters; two of them are VSI inverters and the other two are PQs. The zone definition is based on the sensitivity of each bus voltage to the power produced by each individual inverter when a load increase equal to one unit occurs at that bus. That means if there is one unit of load change at a particular bus, the bus will face a voltage drop. This voltage drop can be compensated by specific amount of power from each DG once at a time. The DG that is capable of bringing the voltage back to its nominal value with generating less power will include that bus in its own zone. In this case, the voltage drop can be compensated with less effort or in other words with generating less power. As discussed, there are 4 inverters attached to the microgrid so there will be 4 zones inside the microgrid (since each bus is more sensitive to one of the inverters, the number of zones would be equal to the number of inverters).

**Fig. 6:**
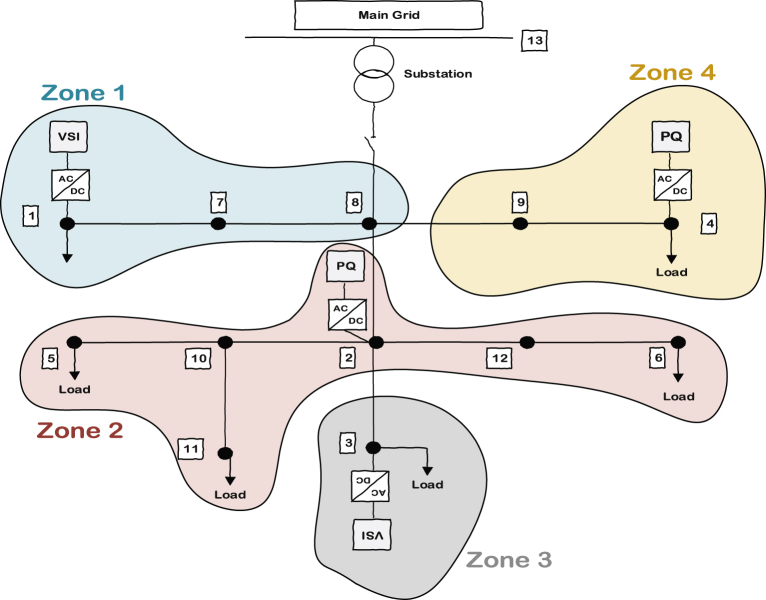
Zone dedication to PQ and VSI inverters considering the bus voltage sensitivity to the power production of inverters.

Fig. 6 shows how these zones should include different buses of the system to improve the microgrid's voltage quality. The problem formulation related to sensitivity analysis of zone dedication is discussed in the next section. When a zone includes a specific bus, it means that it will be better to support the added load to that bus by its corresponding inverter in order to get smoother voltage profile.

The zone dedication approach will help improving the voltage profile by reducing the variance of nodes voltages from the desired average value. In fact, by implementing ACA on VSIs, the average voltage of nodes will be maintained at the desired average value. However, the variance of bus voltages from the average value may not be acceptable in some situations and it is possible that some nodes voltages deteriorate the minimum and maximum thresholds of the voltage profile.

The implemented droop-based primary and consensus-based average voltage control in secondary of VSI inverters make them responsible for all the load changes in the system no matter where it is located. They all participate to supply the demanded active and reactive power and keep the average voltage at the desired level. This control is necessary and inevitable because it helps to avoid voltage and frequency instability. In other words, each islanded microgrid needs to have at least one inverter working at VSI mode and working in this mode means that they do not decide how much power they are producing; instead, they have to produce power until the voltage and frequency reach the predefined states. So, in order to implement the aforementioned zoning scheme, only PQ inverters have to take actions when they see a demand in their zone. It should be noted that each PQ will understand the power demand inside its territory by measuring the power flow of tie lines which connect it to other zones.

**Fig. 7:**
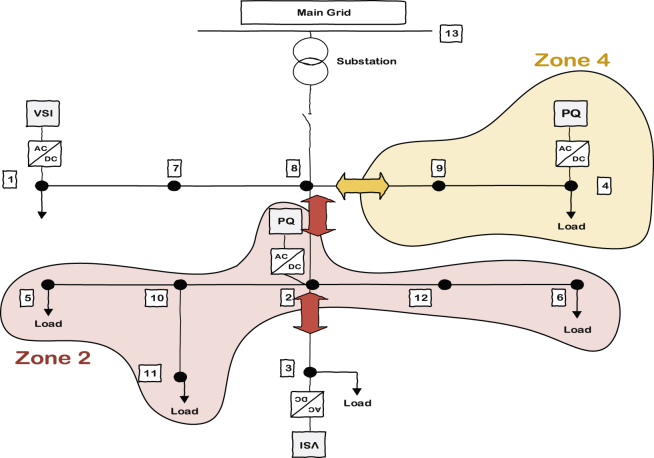
Remained zones related to PQs along with their tie lines.

Based on the above discussion, it is clear that VSI inverters do not need to measure their tie lines flow and if only PQ inverters do their job regarding the supply of the demanded load in their own areas, VSI inverters will automatically supply the rest of microgrid. This fact is shown in Fig. 7 by removing the VSIs zone definition from the picture. It can be also seen that zone 4 has only one tie line to consider while zone 2 has 2 tie lines.

## 4 Analysis

### 4.1 Sensitivity analysis for zone definitions

In this section, bus voltage sensitivity is analyzed in microgrid to achieve better voltage regulation in case of any changes in the load demand. One of the most common methods in the literature such as [13] is Jacobian-Based method. In this method, first the load flow is solved and the values of P and Q are obtained. Then the inverse of Jacobian matrix at that operating point is used to find the sensitivity of the system. The procedure is described in (Eqs. 15 and 16).

(15) $$\left[\begin{array}{l} \Delta \delta \\ \Delta V \end{array}\right]=\left[\begin{array}{l} \begin{array}{cc} \left[S_{\delta p}\right] & \left[S_{\delta q}\right] \end{array}\\ \begin{array}{cc} \left[S_{vp}\right] & \left[S_{vq}\right] \end{array} \end{array}\right].\left[\begin{array}{c} \Delta P\\ \Delta Q \end{array}\right]$$

(16) $$\Delta V=S_{vp}\Delta P+S_{vq}\Delta Q$$

Since *S*_*vp*_ is mostly smaller than *S*_*vq*_, the first term can be ignored to use the simpler format which is

(17) $$\Delta V=S_{vq}\Delta Q$$

In other words, we could find the effect of changing ΔQ on ΔV from the matrix of *S*_*vq*_. However, this method is not always working very well since it is dependent on the load flow calculations and operating points as well. In fact, if the Jacobian matrix is ill-conditioned which is the case in most distribution systems, then, it is hard to find the solution of power flow by using Newton-Raphson method. That's why here an improved method of finding the sensitivity analysis in the distribution system is introduced and used.

#### 4.1.1 Improved method based on fast decoupled load flow

Fast Decoupled Load Flow (FDLF) is chosen for sensitivity analysis in this paper. FDLF analysis utilizes the assumption of having inductive grid in order to makes the analysis simple. However, considering the fact that the exact sensitivity values are not going to be used in this study, but it will be used just as an indicator or tool to assign a zone to each bus (determine if the bus is or is not part of a zone), makes it possible to expand the result to microgrids and not deteriorating the result. That's why the FDLF-based sensitivity analysis which provides an estimation of the sensitivity will work here.

Finding sensitivities based on this method is very quick and also it is not dependent on the operating point. These are two important reasons for using this method. In this method, the Jacobian matrix is only derived once and it is not needed to be calculated for each iteration. We can find the Jacobian Matrix in Fast Decoupled Load Flow as follows:

(18) $$\left[\begin{array}{cc} \left[\begin{array}{l} \frac{\partial f_{pk}}{\partial \delta_{k}}=\sum_{\begin{array}{l} i=1\\ i\neq k \end{array}}^{N}V_{k}B_{ki}\\ \frac{\partial f_{pk}}{\partial \delta_{i}}=-V_{k}B_{k}\left(i\neq k\right) \end{array}\right] & \left[\begin{array}{l} \frac{\partial f_{pk}}{\partial V_{k}}=0\\ \frac{\partial f_{pk}}{\partial V_{i}}=0\left(i\neq k\right) \end{array}\right]\\ \left[\begin{array}{l} \frac{\partial f_{pk}}{\partial V_{k}}=0\\ \frac{\partial f_{pk}}{\partial \delta_{i}}=0\left(i\neq k\right) \end{array}\right] & \left[\begin{array}{l} \frac{\partial f_{Qk}}{\partial V_{k}}=-2V_{k}B_{kk}-\sum_{\begin{array}{l} i=1\\ i\neq k \end{array}}^{N}V_{i}B_{ki}\approx V_{k}\left(-2B_{kk}-\sum_{\begin{array}{l} i=1\\ i\neq k \end{array}}^{N}B_{ki}\right)\\ \frac{\partial f_{QK}}{\partial V_{i}}=-V_{i}B_{ki}\approx -V_{k}B_{ki}\left(i\neq k\right) \end{array}\right] \end{array}\right]$$

As it can be seen, the off-diagonal elements of the Jacobian matrix are zeros based on the FDLF assumptions. The diagonal elements can be rewritten so that two new parameters called $B^{\prime }$and $B^{\prime \prime }$are defined in (Eqs. 19 and 20). Then, the simpler format for Jacobian matrix will be obtained in (Eq. 21).

(19) $${B^{\prime }}_{kk}=\sum_{i=1,\;i\neq k}^{N}B_{ki};{B^{\prime }}_{ki}=-B_{ki}\left(i\neq k\right)$$

(20) $$B^{\prime \prime }=-2B_{kk}-\sum_{i=1,i\neq k}^{N}B_{ki};\;\;\;{B^{\prime \prime }}_{ki}=-B_{ki}\left(i\neq k\right)$$

(21) $$\left[\begin{array}{cc} \left[\begin{array}{l} \frac{\partial f_{pk}}{\partial \delta_{k}}=\sum_{\begin{array}{l} i=1\\ i\neq k \end{array}}^{N}V_{k}B_{ki}=V_{k}\sum_{\begin{array}{l} i=1\\ i\neq k \end{array}}^{N}B_{ki}=V_{k}{B^{\prime }}_{kk}\\ \frac{\partial f_{pk}}{\partial \delta_{i}}=-V_{k}B_{ki}=V_{k}{B^{\prime }}_{ki}\left(i\neq k\right) \end{array}\right] & \left[0\right]\\ \left[0\right] & \left[\begin{array}{l} \frac{\partial f_{QK}}{\partial V_{k}}=V_{k}\left(-2B_{kk}-\sum_{\begin{array}{l} i=1\\ i\neq k \end{array}}^{N}B_{ki}\right)=V_{k}{B^{\prime \prime }}_{ki}\\ \frac{\partial f_{Qk}}{\partial V_{i}}=-V_{k}B_{ki}=V_{k}{B^{\prime \prime }}_{ki}\left(i\neq k\right) \end{array}\right] \end{array}\right]$$

Now, by defining *V* as in (Eq. 22), $\frac{\partial f_{Qk}}{\partial V_{i}}=V_{k}{B^{''}}_{ki}\;$can be rewritten in a matrix format as shown in (Eq. 23).

(22) $$\left[V\right]=\left[\begin{array}{cccc} V_{1} & 0 & 0 & 0\\ 0 & V_{2} & 0 & 0\\ 0 & 0 & ... & 0\\ 0 & 0 & 0 & V_{n} \end{array}\right]\;$$

(23) $$\left[\frac{\partial f_{Q}}{\partial V}\right]=\left[V.B^{\prime \prime }\right]$$

Zones sensitivities are defined as percentage increase in the voltage of a bus when approximately 1 extra unit of reactive power is injected to the microgrid by an inverter with respect to inverters bus voltage. This definition is shown in (Eq. 24).

According to the definition of zone sensitivity and by rearranging (Eq. 23), it can be seen in (Eq. 25) that$B^{\prime \prime }$matrix elements are describing the inverse of zone sensitivities. So zone sensitivities can be described by the inverse of $B^{\prime \prime }$matrix as in (Eq. 26).

(24) $$\text{Zone}\;\text{sensitivity}=\frac{\partial V_{i}}{\;\;\;\;\;\;\frac{\partial f_{Qk}}{V_{k}}\;\;\;\;\;}$$

(25) $$B^{\prime \prime }=V^{-1}.\left[\frac{\partial f_{Qk}}{\partial V_{i}}\right]=\left[\frac{\frac{\partial f_{Qk}}{V_{k}}}{\;\;\;\;\;\;\partial V_{i}\;\;\;\;\;\;\;\;\;}\right]$$

(26) $$\text{Zone}\;\text{sensitivity}=\frac{\partial V_{i}}{\frac{\partial f_{Qk}}{V_{k}}}={B^{\prime \prime }}^{-1}$$

Here, the procedure is explained in details. In transmission system's load flow and sensitivity analysis, one of the generator buses is treated as slack bus to carry the entire loss of the system which is kept out of calculation during iterations. In microgrids, we do not have a real slack bus due to the characteristics of the microgrid. However, in the proposed approach, the zone definition analysis considers a virtual slack bus. The analysis needs to be repeated several times (for 4 DG it needs to be repeated 4 times) and each time one of the DGs buses will be chosen to be the slack, then with respect to this assumption the zones will be defined.

In Table 1, the result of zone definition analysis is shown when bus 1 (connected to DG1) is considered to be the slack. So other buses are assigned to the zones of DG2, DG3, and DG4 since the row and column of the DG1 is eliminated. Consequently, just by looking at this table the decision about the accurate zones of buses cannot be made because the buses which should be in the zone of DG1 are now assigned to other three zones. That's why you cannot see zone one in Table 1 which consider DG1 to be the slack. In other words, when a virtual slack bus is considered, instead of 4 zones only 3 zones are formed and therefore we require this analysis to be repeated for each DG as slack one separately.

**Table 1:** Zone Calculation Considering **Bus 1 as Slack**.

Bus #	2	3	4	5	6	7	8	9	10	11	12
Zone	2	3	4	2	2	4	4	4	2	2	2

As mentioned earlier, we need to repeat this process by assuming another inverter bus to be the slack bus. So, for example by assuming bus 2 as the new slack bus, we can find out which buses are sensitive to bus 1, 3, and 4; but again the buses that are sensitive to bus 2 are not specified this time.

In other words, the result of sensitivity for each iteration does not reflect the effect of the eliminated bus in the defined zones. Therefore, to solve this problem, we need to repeat this process until all of the inverter buses are once assigned to be the slack bus. Then, by looking at each bus individually the most repeated zone in different cases can be determined and recognized as the accurate zone. For example, for bus 5, zone 2 is repeated the most which shows that it is more sensitive to DG 2. The total result is shown in the last row of Table 2.

**Table 2:** Zone Calculation Considering each inverter bus as slack once.

Bus #	1	2	3	4	5	6	7	8	9	10	11	12
Zones (Bus 1 is eliminated)	–	2	3	4	2	2	4	4	4	2	2	2
Zones (Bus 2 is eliminated)	1	–	3	4	1	1	1	1	4	1	1	1
Zones (Bus 3 is eliminated)	1	2	–	4	2	2	1	1	4	2	2	2
Zones (Bus 4 is eliminated)	1	2	3	–	2	2	1	1	1	2	2	2
Accurate Zones	1	2	3	4	2	2	1	1	4	2	2	2

## 5 Results & discussion

**Fig. 8:**
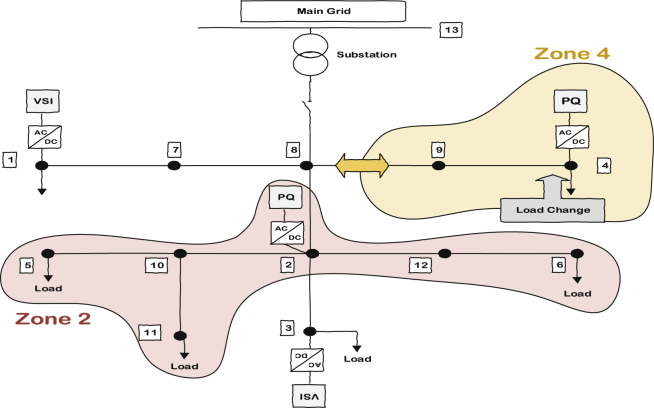
Load change location in case one and two.

The islanded 13 bus microgrid is used for verifying the effectiveness of zone dedication in reducing the variance of nodes voltages from the desired average value. Fig. 8 shows the single line diagram of the microgrid test system along with the dedicated zones to PQ inverters. The nominal frequency and voltage are 50 Hz and 380 V, respectively. The loads are modeled as typical RL loads through cases 1–4 and in the last case they are replaced with voltage dependent loads to verify the effectiveness of the control scheme for different type of loads. RL branches are also used for the lines between buses. Bus 13 in Fig. 8 shows the point of common coupling. Simulation results are presented in four different scenarios. In the first two cases, the load change occurs in zone 4; it is a demand increase for case one and then a demand decrease for case two. In case three, the demand change will happen in zone 2. In all cases, the average voltage of main buses (PQ buses, VSI buses and critical load buses including bus 5 and 6) are regulated to the predetermined peak value of 456 Volt or rms value of 323.4 Volt by using ACA at VSI layer. It is assumed that a 2% mismatch from the average is acceptable.

The result can demonstrate that only by using ACA control for VSI layer, the average voltage can be regulated effectively; however, the minimum and maximum requirement might not be met just by involving the VSIs.

Finally, case 4 performs delay response analysis due to the existence of possible time delays in communication links of VSI or PQ layer.

### 5.1 Case one

In first scenario, the islanded microgrid faces a demand increase in bus 4 located in zone 4. The line between bus 8 and 9 is the only tie line of this zone and its power flow should be considered. The DG attached to bus 4 communicates with the agent located at the mentioned tie line to sense the power flow and change the PQ inverter's set-point based on that. In this scenario, the active and reactive load increases are equal to 4.5 k watt and 5 kvar respectively and it happens at t = 1.5 second. The results in absence and presence of zones are presented here.

**Fig. 9:**
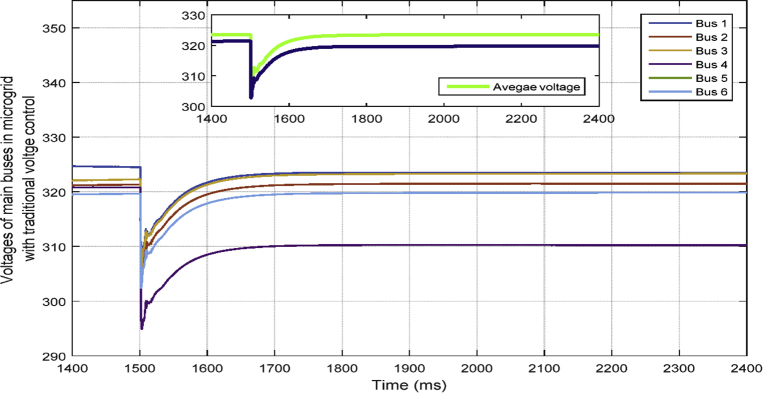
Voltages of main 6 buses with traditional voltage control in case 1.

Fig. 9 shows the six main bus voltages when the traditional voltage control is applied. In traditional secondary voltage control, only the voltages of VSI buses are controlled and fixed at a specific set-point—for example, 323.4 rms.

**Fig. 10:**
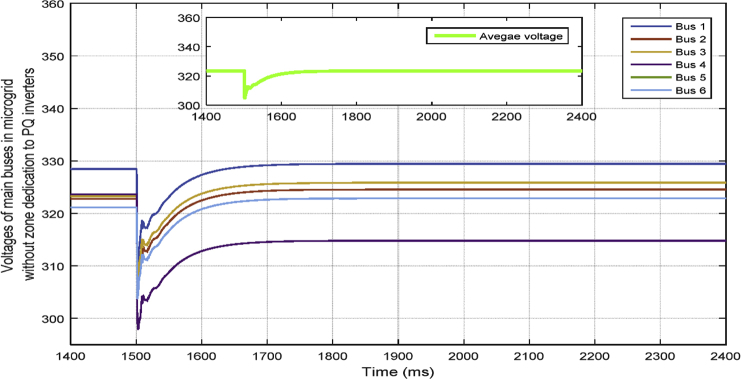
Voltages of main 6 buses with ACA-based voltage control in case 1.

**Fig. 11:**
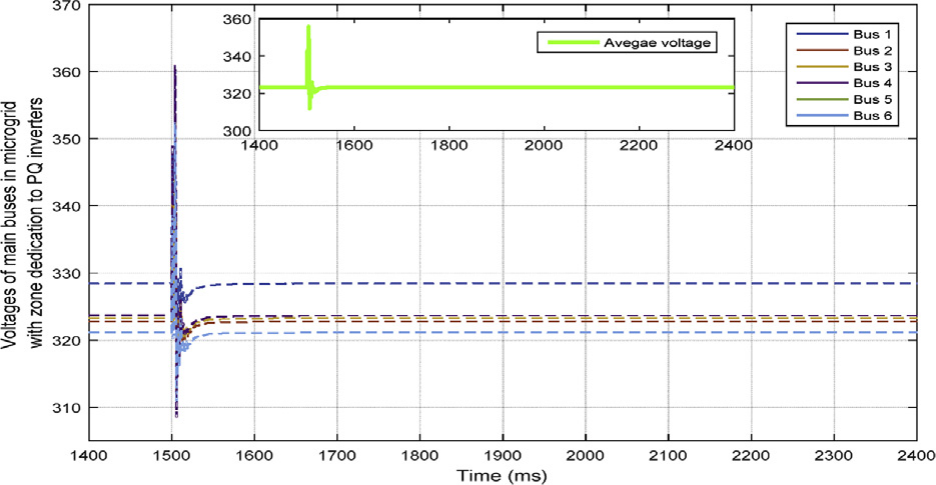
Voltages of main 6 buses with ACA-based voltage control for VSIs along with zone dedication to PQ inverters in case 1.

In Fig. 10, the consensus-based average voltage control is implemented through VSI inverters. In this situation, PQ inverters are not involved yet. It can be seen from the result that the voltage at bus 4 experiences a drop due to the load change even though the average voltage regulator is doing its job correctly. Later, by dedicating zones to PQs, they will contribute in voltage control and the result of this involvement is shown in Fig. 11. PQ inverters collect information from tie line agents and make sure that the load demand in their own area is fed through themselves, not any other inverter.

**Fig. 12:**
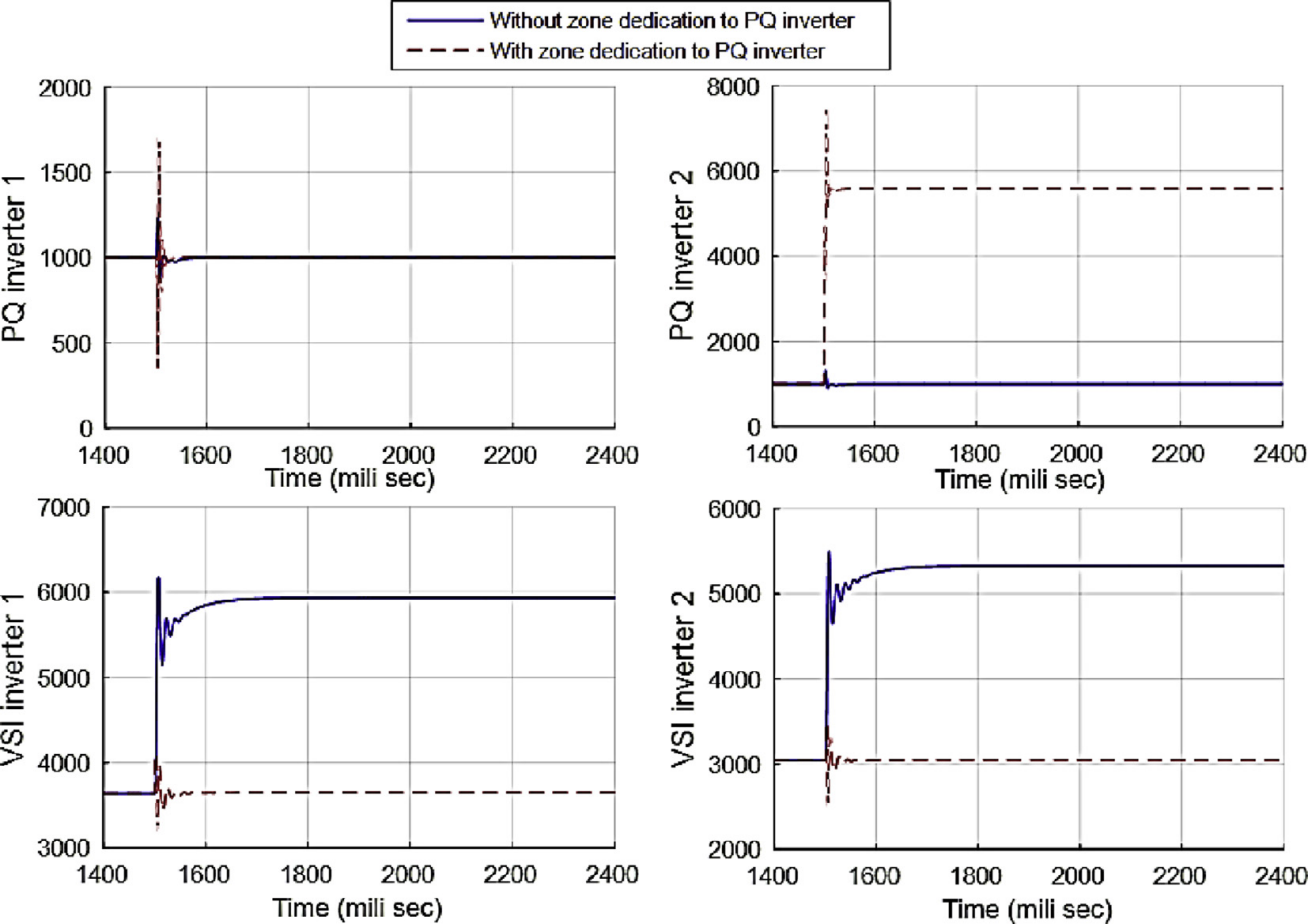
Active power produced by inverters in case 1.

**Fig. 13:**
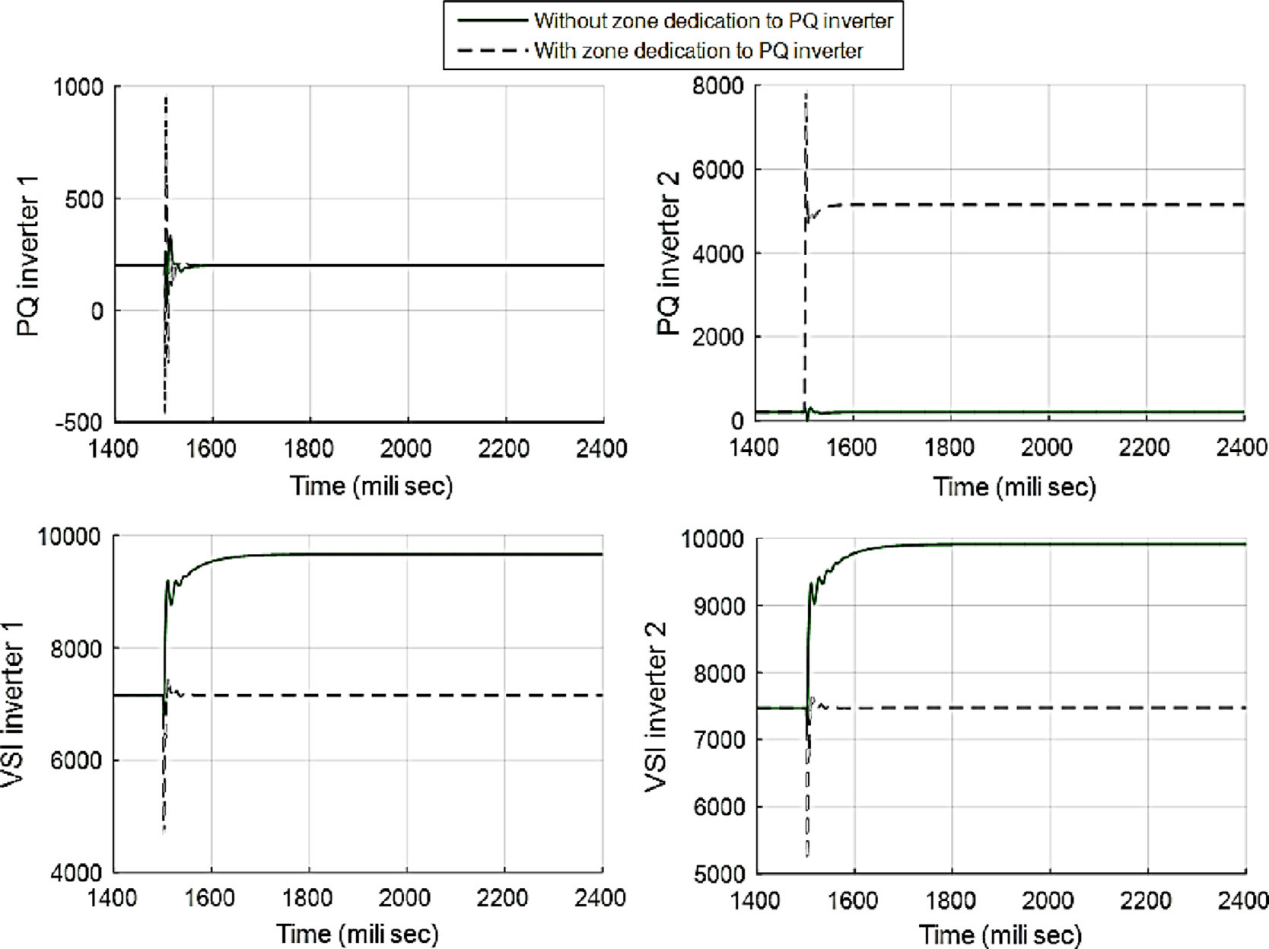
Reactive power produced by inverters in case 1.

**Fig. 14:**
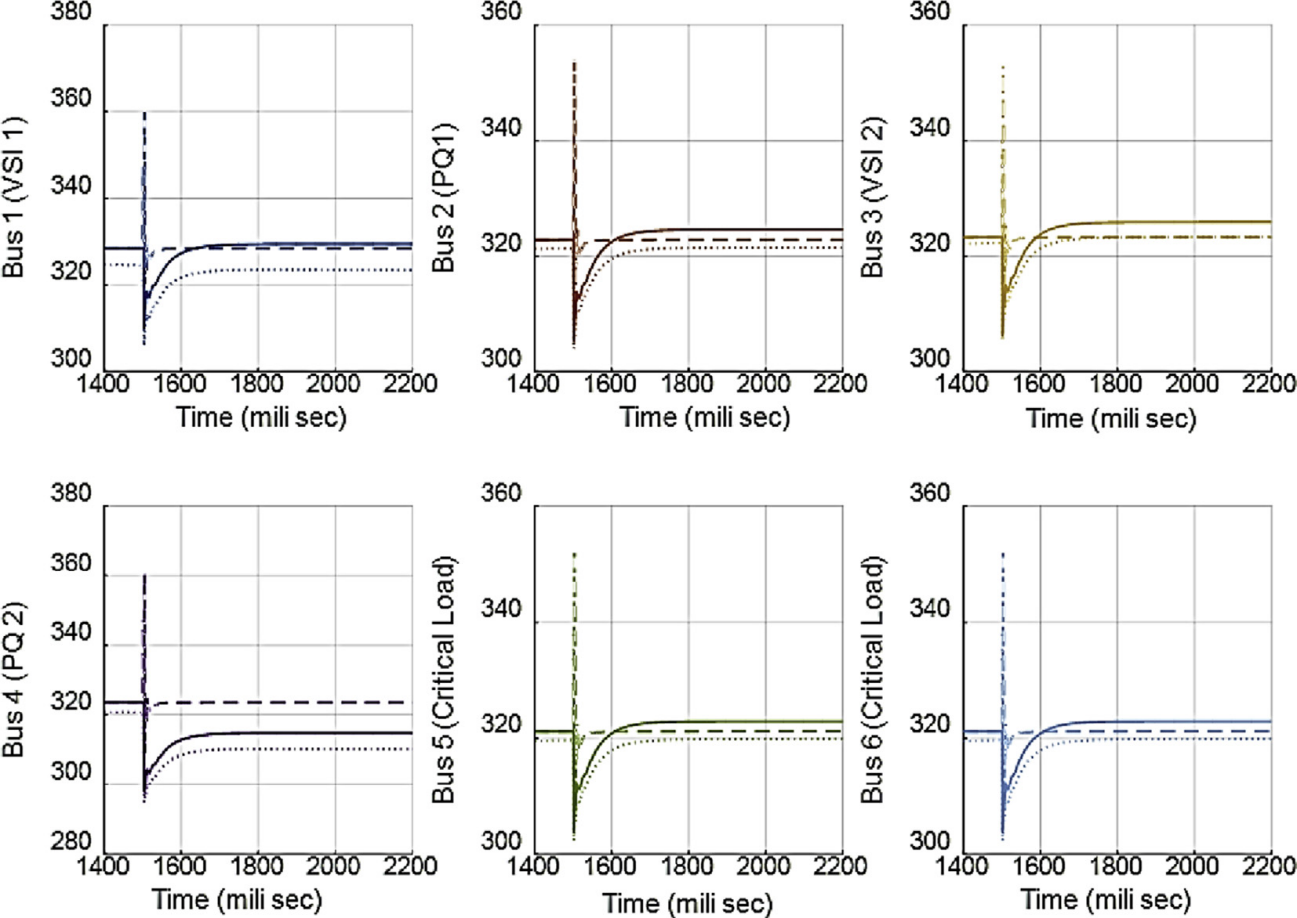
Main bus voltages under different controllers-traditional (Doted), ACA-based (Solid) and zone dedicated method (Broken).

This fact can be seen in Figs. 12 and 13, which show the active and reactive power produced by the 4 available inverters in the microgrid. Before implementing the zone definition method, both PQ inverters are providing constant power and the other two VSI inverters are taking care of the load demand; however, after giving the responsibility to the PQ inverter attached to bus 4, it is going to be the only inverter changing its output due to demand's increase. Fig. 14 compares the voltages one-by-one to demonstrate the effect of all three controllers on bus voltages.

**Fig. 15:**
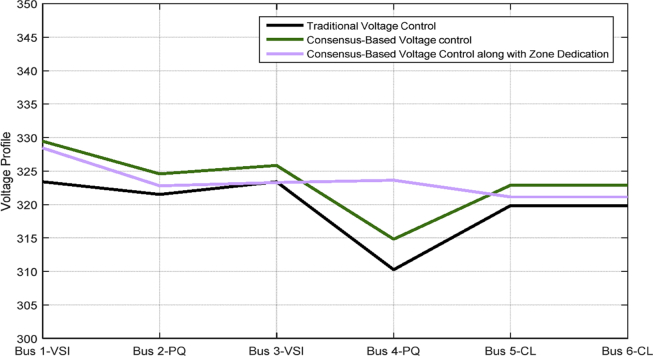
Voltage profile of main buses with three different control scheme (traditional, ACA-based and zone dedicated method) in case 1.

Table 3 shows the steady state voltages of all six buses using three different control schemes including traditional secondary controller and consensus-based controller with and without zone dedication. The mean value and variance are calculated for all circumstances. It can be seen that the variance of voltages is significantly reduced through the contribution of PQs in supplying the load demand comparing to ACA-based control alone. Voltage profile is shown in Fig. 15 which demonstrates the mentioned table on a graph.

**Table 3:** Steady state voltage value with and without zone dedication to PQ inverters for load increase in zone 4.

	V_1_	V_2_	V_3_	V_4_	V_5_	V_6_	Mean	Variance
Traditional control	323.4	321.5	323.4	310.25	319.83	319.83	319.70	120.2
ACA-Based	329.44	324.56	325.85	314.81	322.87	322.87	**323.40**	118.179
With zone dedication	328.44	322.81	323.27	323.64	321.12	321.12	**323.40**	36.16

### 5.2 Case two

**Fig. 16:**
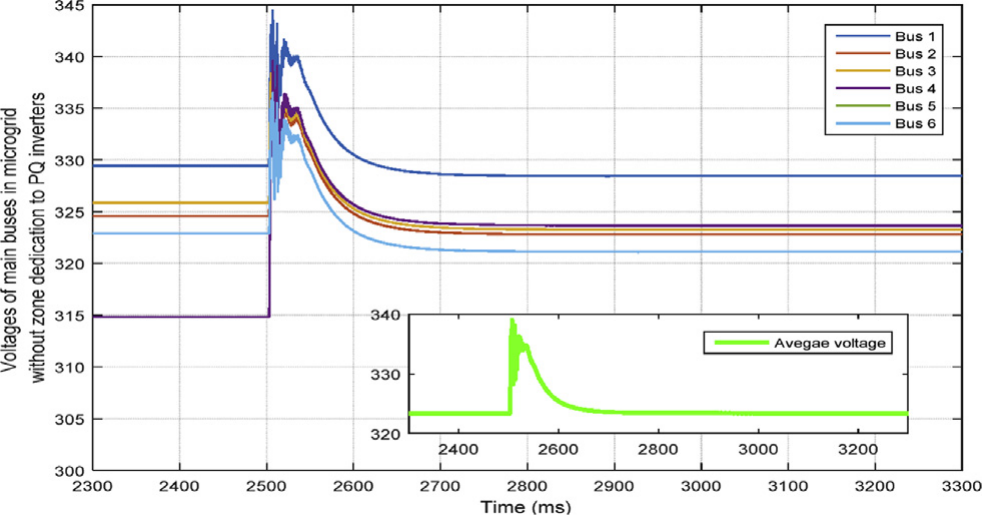
Voltages of main 6 buses with ACA-based control in case 2.

**Fig. 17:**
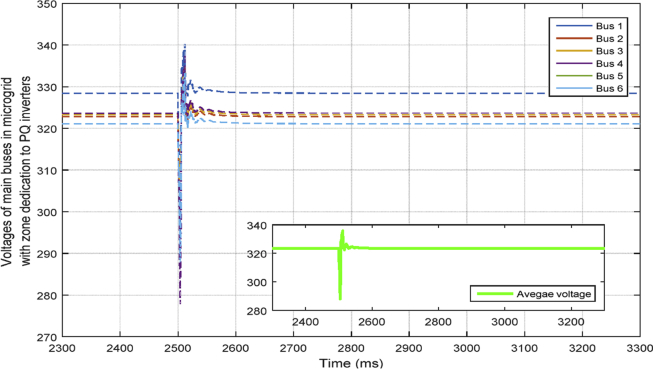
Voltages of main 6 buses with consensus-based voltage control along with zone dedication to PQ inverters in case 2.

Second case is similar to the first case. The only difference is that the load increase is changed to load decrease. The purpose of this scenario is examining the applicability of zone dedication when the load demand is decreased by the same value as case one. In this scenario, the active and reactive load decrease is equal to 4.5 kwatt and 5 kvar respectively and it happens at t = 2.5 sec. The results are presented in absence and presence of zones respectively in Figs. 16 and 17.

**Fig. 18:**
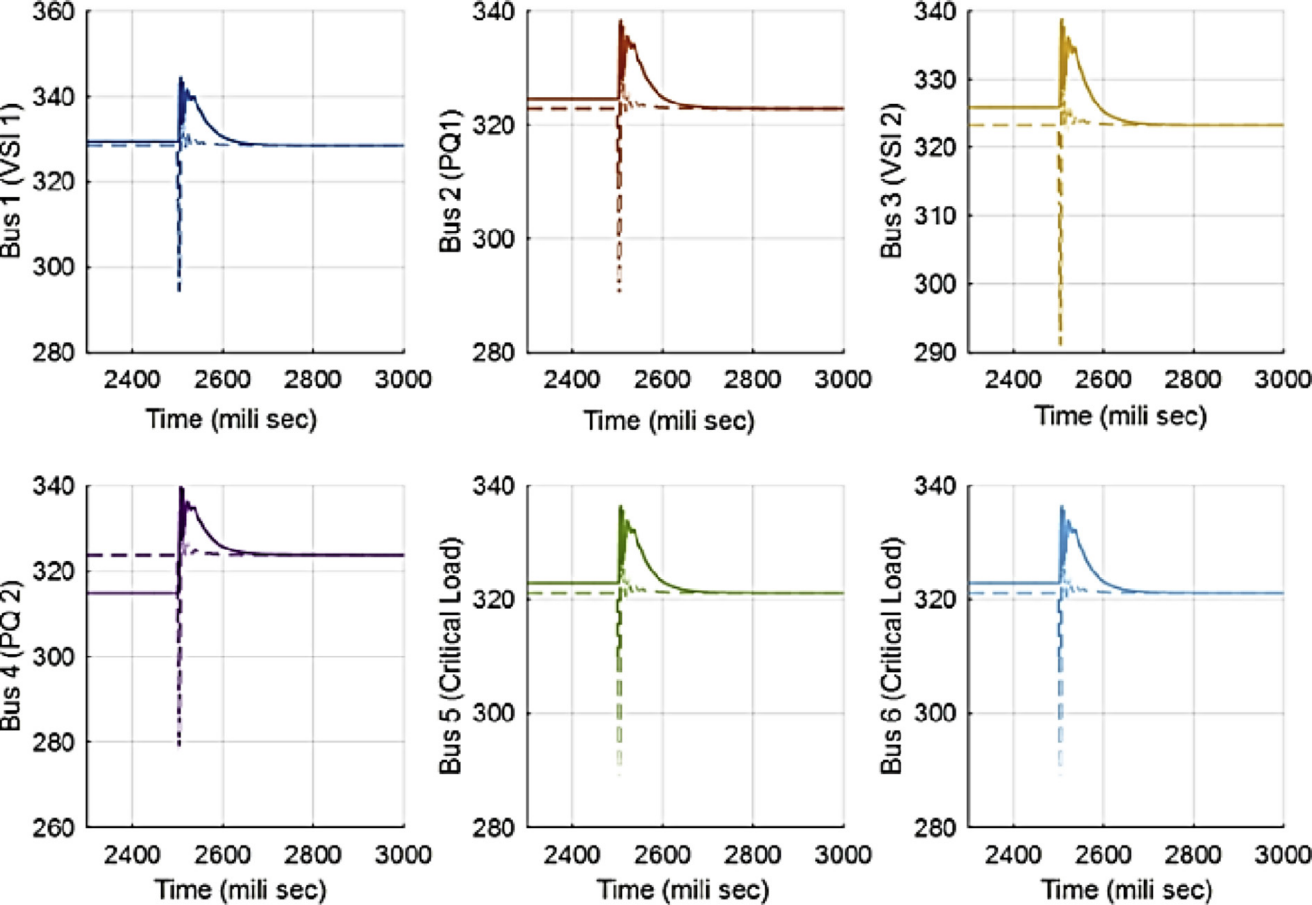
Main bus voltages under different controllers-traditional (Doted), ACA-based (Solid) and zone dedicated method (Broken).

**Fig. 19:**
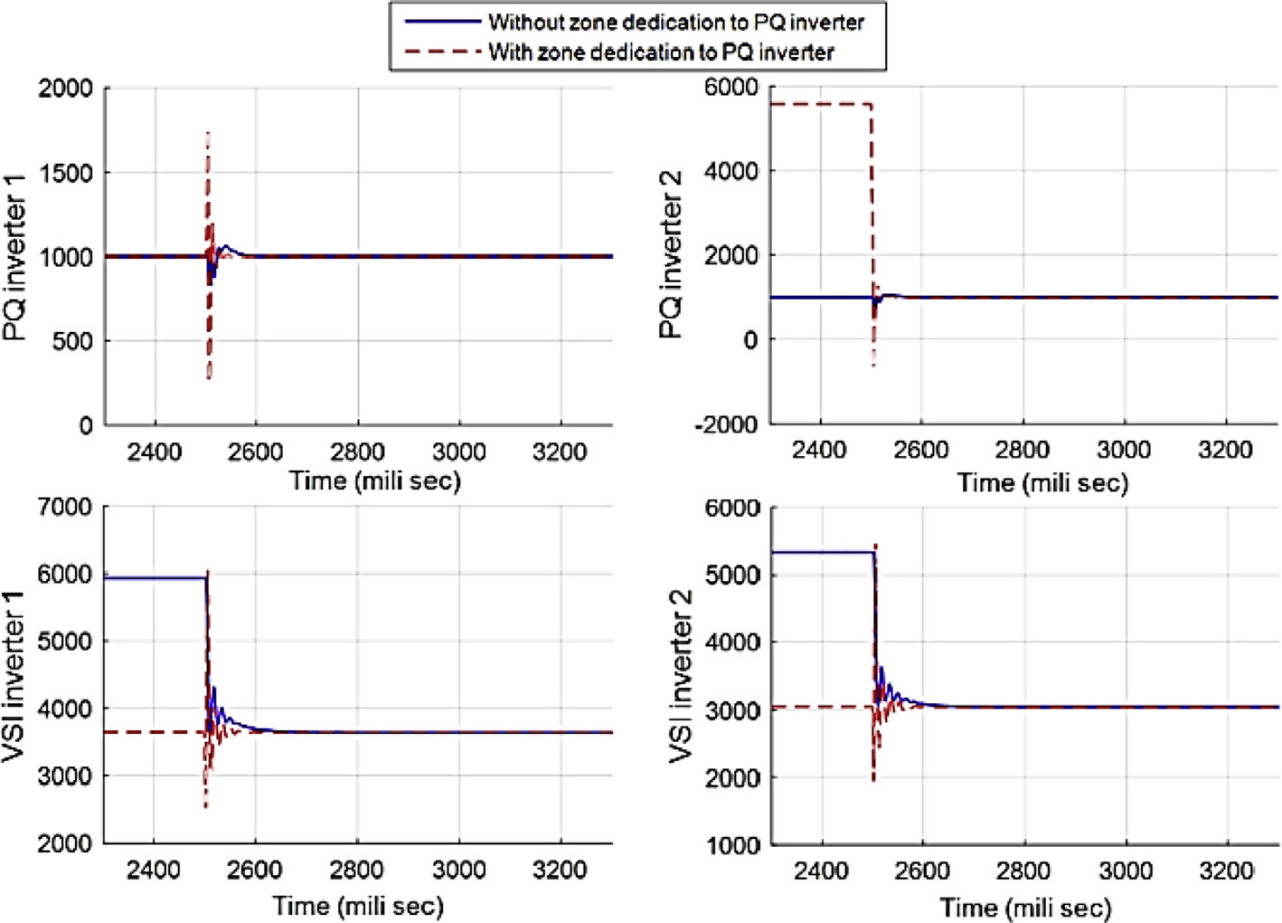
Active power produced by inverters in case 2.

**Fig. 20:**
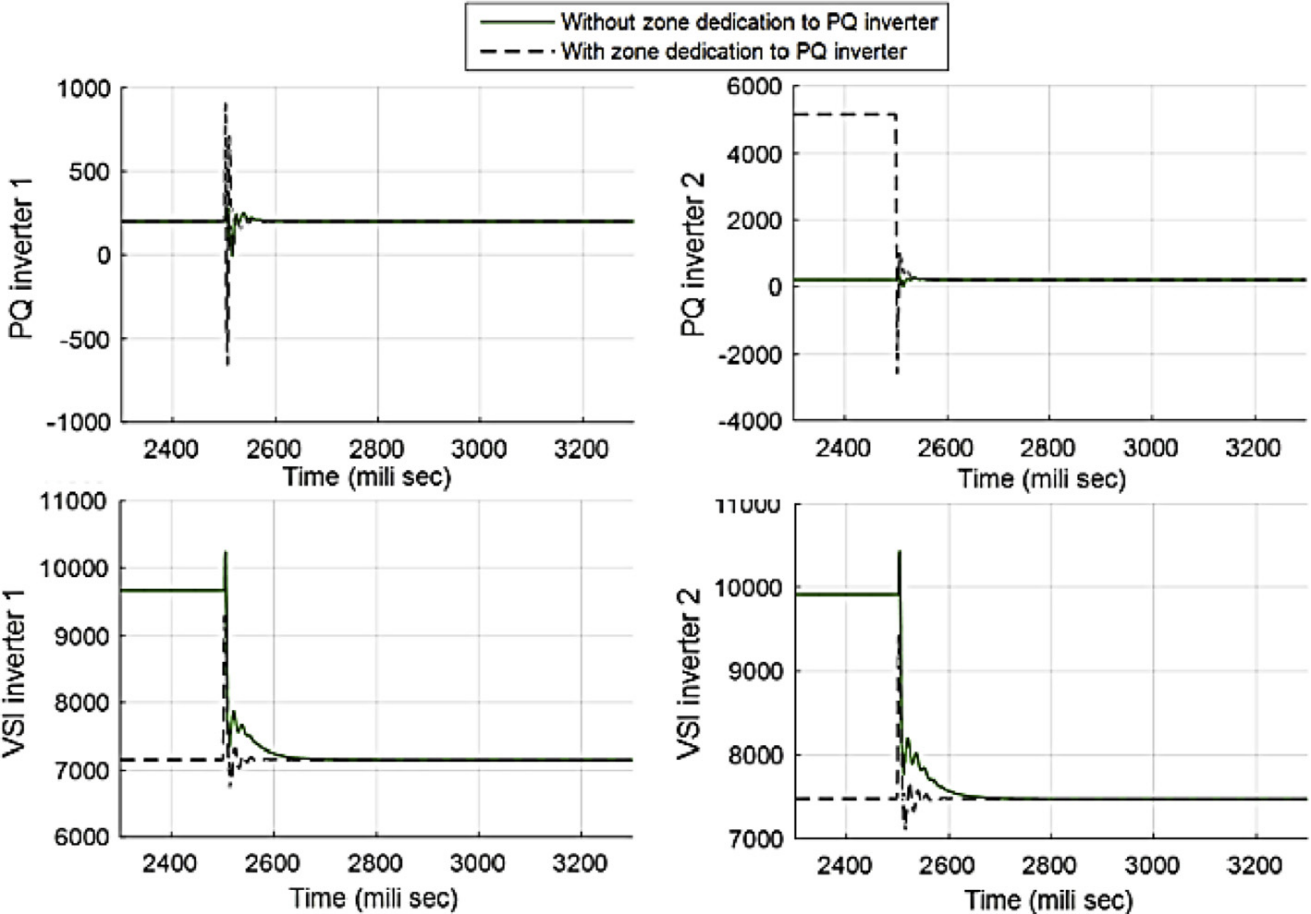
Reactive power produced by inverters in case 2.

Fig. 18 compares the voltages one-by-one. Then, the active and reactive power produced by inverters in the microgrid before and after implementing the zone definition method are shown in Fig. 19 and Fig. 20.

### 5.3 Case three

**Fig. 21:**
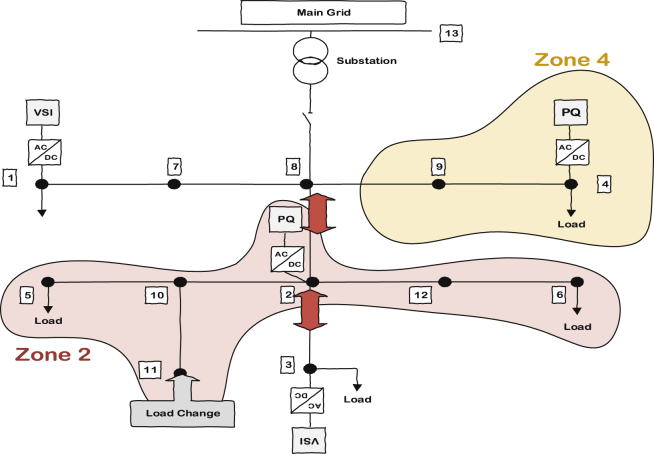
Load change's location in case three.

**Fig. 22:**
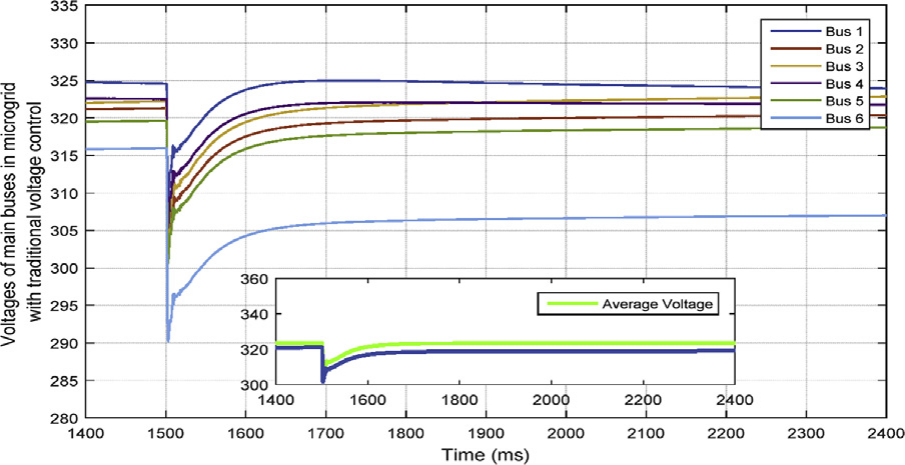
Voltages of main 6 buses with traditional control in case 3.

**Fig. 23:**
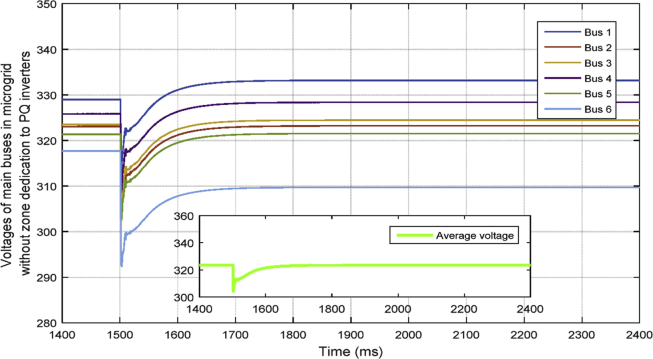
Voltages of main 6 buses with ACA-based control in case 3.

**Fig. 24:**
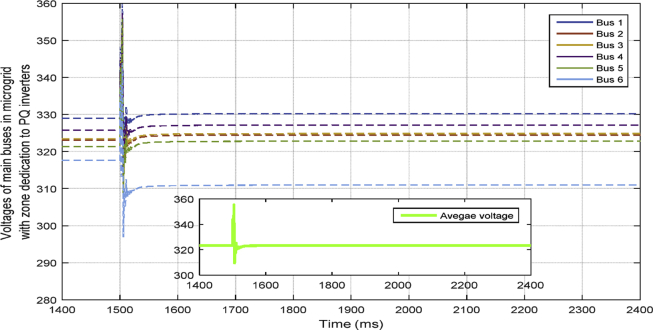
Voltages of main 6 buses with ACA-based voltage control along with zone dedication to PQ inverters in case 3.

The third case is about a load change in zone 2. The difference in this case is related to the distance between the location of the demanded load and the responsible PQ inverter of the zone. In previous cases, the load was attached to the same bus as the PQ inverter; consequently, it had high sensitivity to that specific inverter. However, if there is a long distance between the demanded load and the PQ inverter, the zone dedication might not be as effective as previous cases but it would still be better than the traditional or the consensus-based algorithm. The reason is obvious; the bus voltage with the load change still has the highest sensitivity to the corresponding PQ inverter even though it is not too much. Fig. 21 shows the system and load change scenario inside. This load is added to bus 11 at t = 1.5 sec and then it is disconnected at t = 2.5 sec. The results for all three control scheme—namely traditional control, ACA-based control and ACA-based control with zone dedication to PQ inverters—are presented here in Figs. 22, 23, and 24 respectively.

**Fig. 25:**
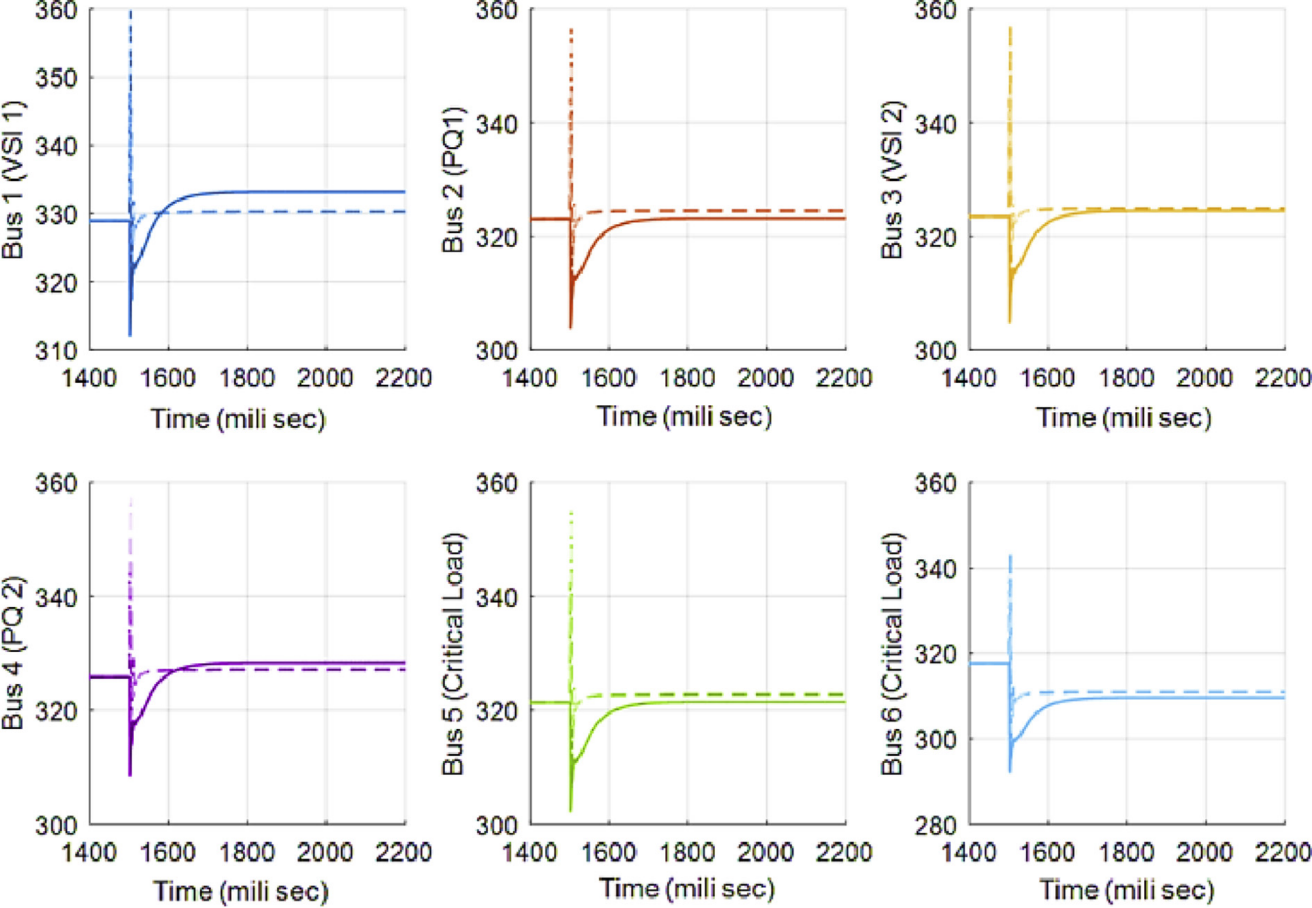
Main bus voltages under different controllers-traditional (Doted), ACA-based (Solid) and zone dedicated method (Broken).

**Fig. 26:**
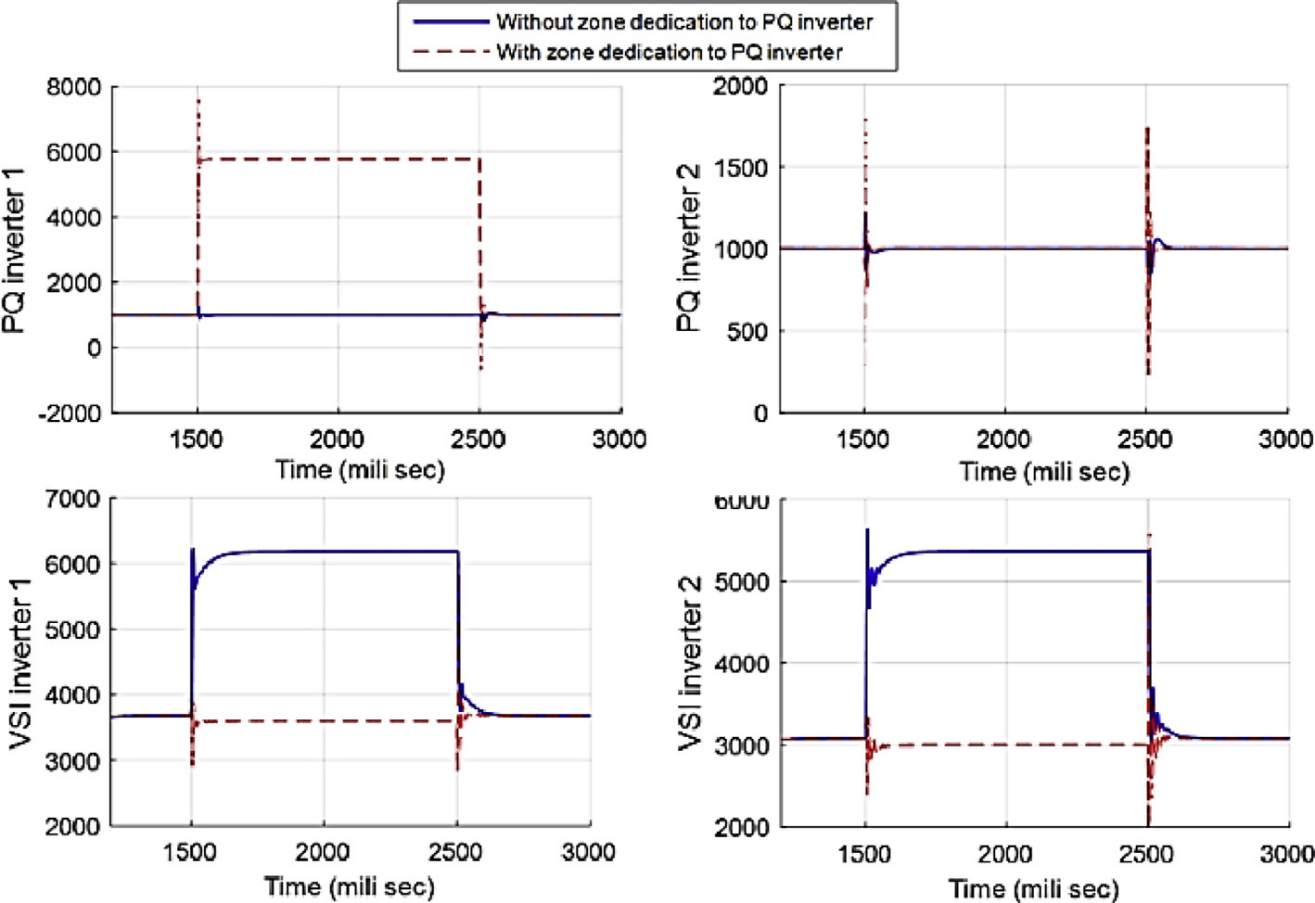
Active power produced by inverters in case 3.

**Fig. 27:**
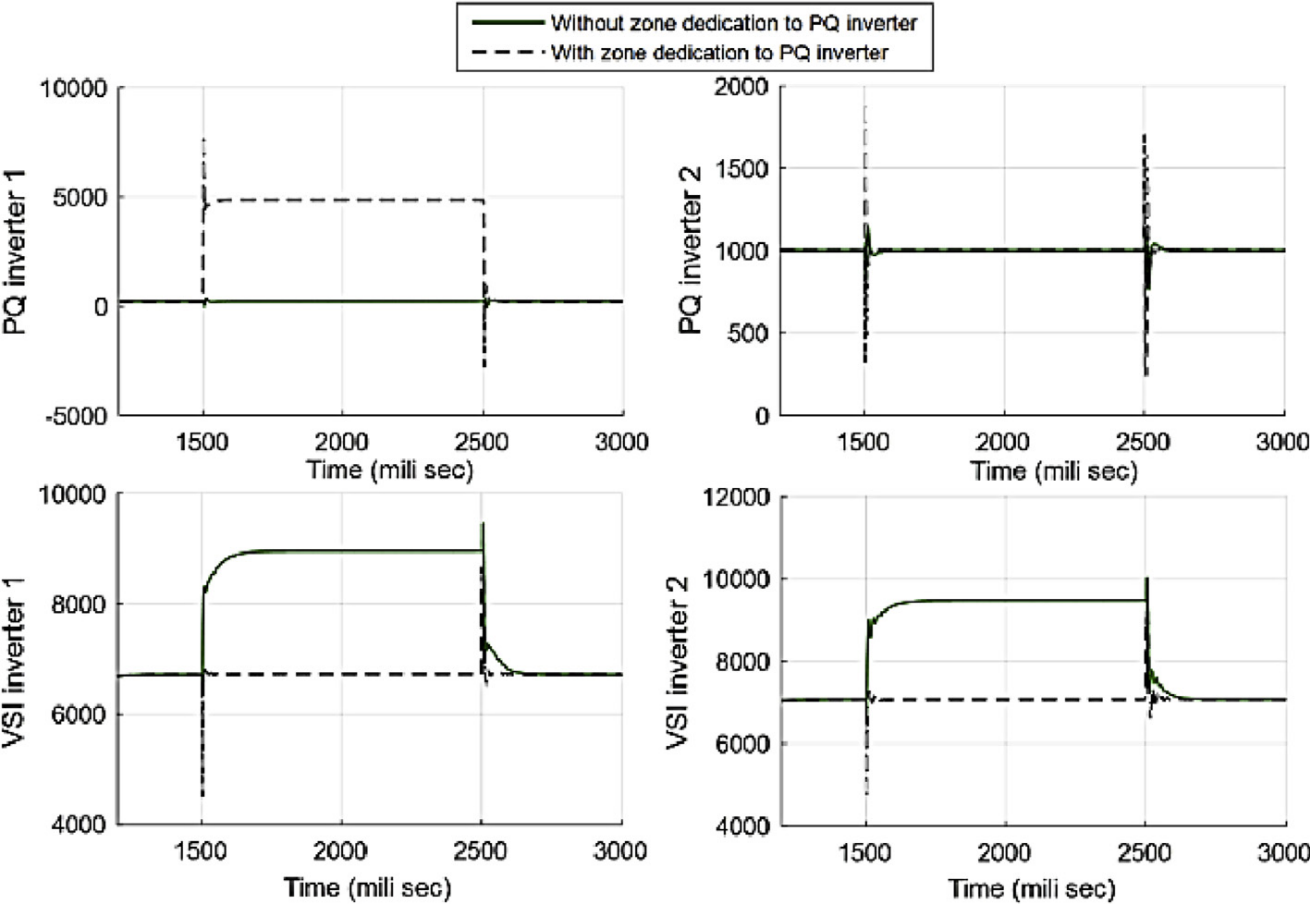
Reactive power produced by inverters in case 3.

Fig. 25 compares the voltages one-by-one to demonstrate the effect of these controllers on voltages. Figs. 26 and 27 show the active and reactive power produced by the inverters in the microgrid before and after implementing zone definition.

Table 4 shows the steady state voltages at all six buses using three different control schemes including a traditional and ACA-based controller with and without zone dedication. The mean value and variance are calculated for all circumstances. By comparing the consensus-based voltage control with and without zone dedication, it can be observed that the variance of voltages is reduced slightly and not as significant as first case, but, it still improves the variance, which means it can increase the lowest voltage of microgrid.

**Table 4:** Steady state voltage value with and without zone dedication to PQ inverters for load increase in zone 2.

	V_1_	V_2_	V_3_	V_4_	V_5_	V_6_	Mean	Variance
Traditional control	323.4	320.42	323.4	321.75	318.7	306.91	319.09	194.5
ACA-Based	333.17	323.19	324.45	328.37	321.52	309.71	**323.40**	312.29
With zone dedication	330.23	324.47	324.86	327.14	322.78	310.94	**323.40**	219.54

**Fig. 28:**
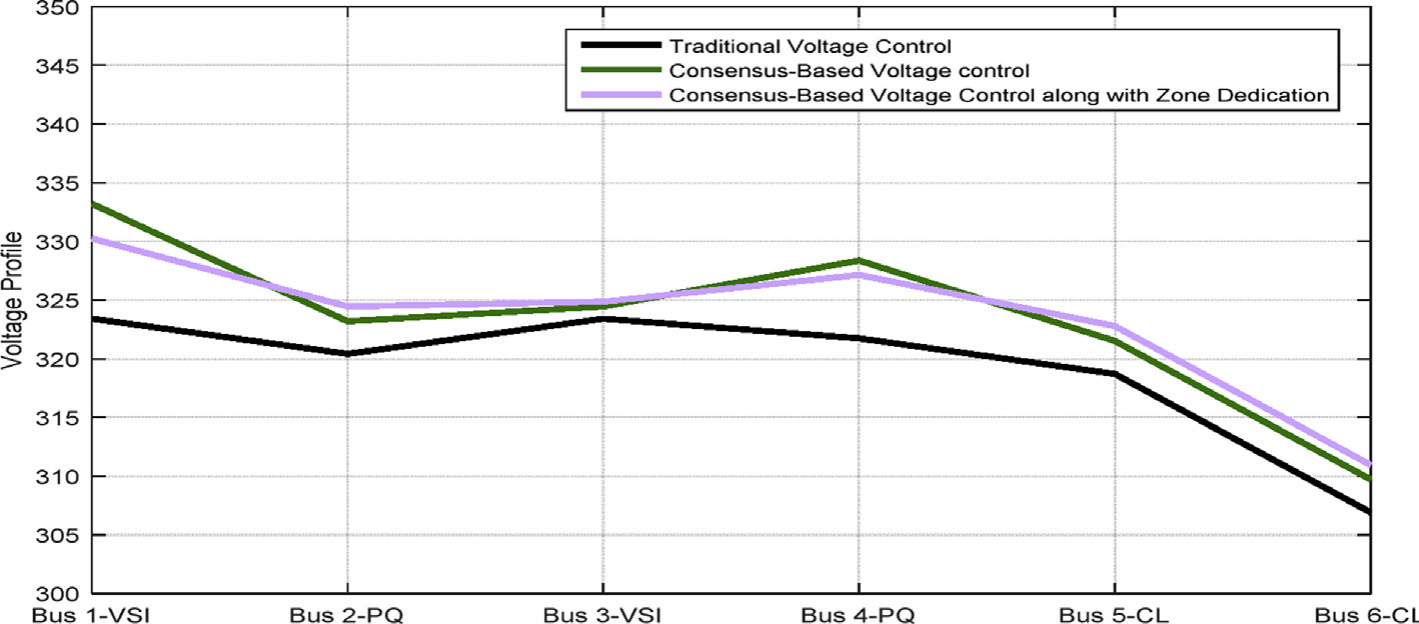
Voltage profile of main buses with three different control scheme (traditional, ACA-based and zone dedicated method) in case 3.

Fig. 28 shows the mentioned table on a graph.

### 5.4 Case four

In this part, the effect of communication delays on simulation results is investigated. Time delay is one of the key uncertainties for online control of systems. Usually, the time delay consists of communication and calculation delays. Here, the negligible calculation time delay in MATLAB can be ignored and Communication time delay can occur in any part of the secondary layer, i.e. VSI or PQ, or both which are analyzed separately in the following three parts.

#### 5.4.1 .Delay in VSI layer (Fixed time delays)

**Fig. 29:**
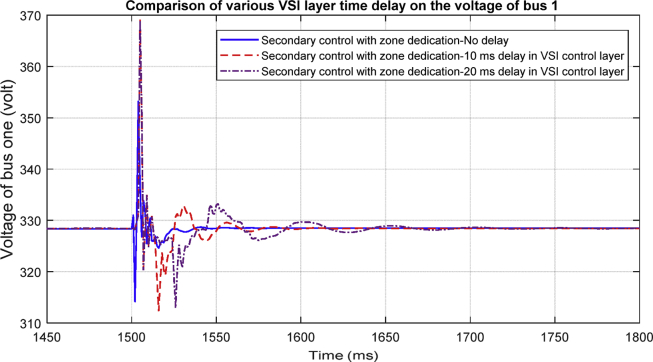
Delay in VSI layer.

Time delay occurs when VSI inverters communicate to reach to an agreement in ACA which is assumed to be a fixed delay here. Fig. 29 shows one of the bus voltages of the system in different scenarios formed to analyze the effect of fixed time delays on the average voltage regulation. A practical delay of approximately 20 milliseconds is considered in this paper and the result shows that the delay does not affect the stability of the system. However, it clearly causes more oscillations, and finally, there would be a threshold after which it will make the controller unstable. So in order to implement this cooperative control, the time-delay constraint needs to be considered and applied.

#### 5.4.2 Delay in PQ layer

**Fig. 30:**
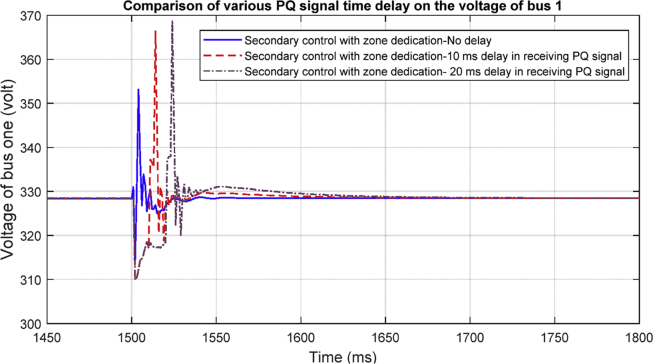
Delay in PQ layer.

Fig. 30 shows the effect of a fixed time delay in PQ layer on the voltage regulation. Again, the results show that the delay of approximately 20 milliseconds does not affect the stability of the system, however the delay in this layer causes slower transient response and a higher overshoot value.

#### 5.4.3 Delay in both VSI and PQ layers

**Fig. 31:**
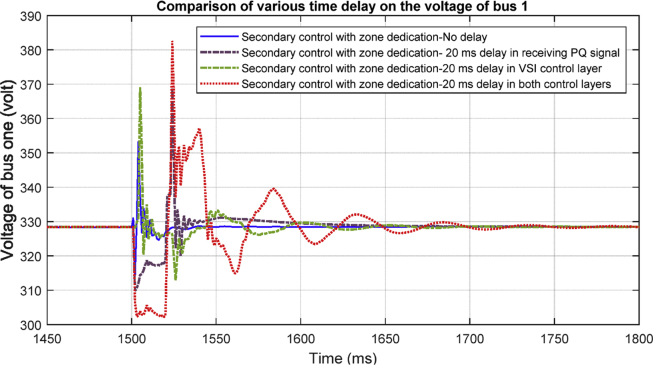
Delay in both layers.

Based on Fig. 31, having delay of 20 milliseconds in both layers of secondary control degrades the transient response of the system significantly, however it does not affect the stability of the system. The result shows that the proposed control is robust against time delays.

### 5.5 Case five

**Fig. 32:**
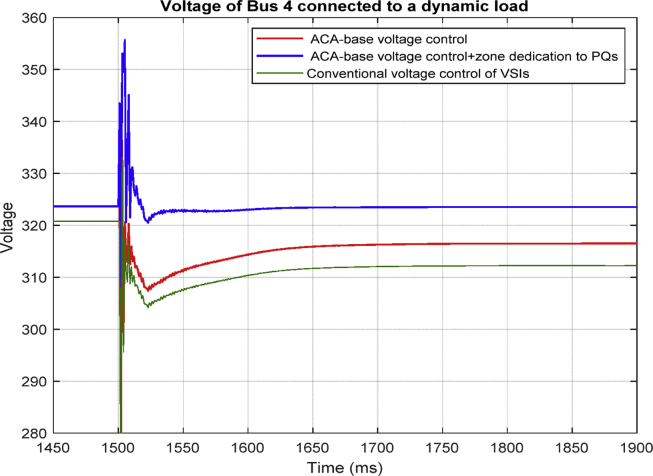
Voltages of bus 4 in different control scheme: conventional and ACA-based voltage control for VSIs with and without zone dedication.

In this scenario, the effect of changing load type on the proposed method is investigated. The RL load model in cased one is replaced with a voltage dependent load and the result is shown in Fig. 32.

By comparing Fig. 11 and Fig. 32 it can be observed that the proposed method works effectively regardless of the load type in the microgrid.

## 6 Conclusion

In this paper, a cooperative secondary voltage control has been proposed in which both VSI and PQ inverters will work together to alleviate the effects of any load change in the system. This cooperative method is based on the zone dedication which is calculated based on the sensitivity analysis to assign each specific bus to a DG in the system.

One of the main contributions of the paper is to improve the steady state response of the system (voltage profile) along with the transient response. The main goal of the paper is steady state response improvement however the strength of the proposed method is that it can improve the transient response as well, and it also has a good margin of stability in case of possible delays in communication signals. The above method has been applied to a microgrid with 13 buses and 4 DGs. The simulation results show the effectiveness of the algorithm in case of different load types and load changes in the system and confirm its robustness in presence of possible time delays in communication signals. A future work can be done by determining tolerable threshold for the delay in communication link in each layer and then analyzing the incoming signals through communication network and enabling a fully decentralized secondary control in case of detection of any delays more than the tolerable threshold for the system to avoid poor performance of the system due to large delays.

## Declarations

### Author contribution statement

Farideh Doost Mohammadi: Conceived and designed the experiments; Performed the experiments; Analyzed and interpreted the data; Contributed reagents, materials, analysis tools or data; Wrote the paper.

Hessam Keshtkar: Conceived and designed the experiments; Analyzed and interpreted the data; Contributed reagents, materials, analysis tools or data; Wrote the paper.

Ali Dehghan Banadaki: Contributed reagents, materials, analysis tools or data; Wrote the paper.

Ali Feliachi: Conceived and designed the experiments.

### Funding statement

This research did not receive any specific grant from funding agencies in the public, commercial, or not-for-profit sectors.

### Competing interest statement

The authors declare no conflict of interest.

### Additional information

No additional information is available for this paper.

## References

[1] Bidram Ali, Davoudi Ali, Lewis Frank L. (2014). A multiobjective distributed control framework for islanded AC microgrids. IEEE Transactions on Industrial Informatics.

[2] Pogaku Nagaraju, Prodanovic Milan, Green Timothy C. (2007). Modeling, analysis and testing of autonomous operation of an inverter-based microgrid. IEEE Trans. Power Electron..

[3] Bidram Ali, Davoudi Ali, Lewis Frank L., Guerrero Josep M. (2013). Distributed cooperative secondary control of microgrids using feedback linearization. IEEE Trans. Power Syst..

[4] Mehrizi-Sani Ali, Iravani Reza (2010). Potential-function based control of a microgrid in islanded and grid-connected modes. IEEE Trans. Power Syst..

[5] Savaghebi M., Jalilian A., Vasquez J.C., Guerrero J.M. (June 2012). Secondary control scheme for voltage unbalance compensation in an islanded droop-controlled microgrid. IEEE Trans. Smart Grid..

[6] Tan K.T., Peng X.Y., So P.L., Chu Y Ch, Chen M.Z.Q. (2012). Centralized control for parallel operation of distributed generation inverters in microgrids. IEEE Transactions on Smart Grid.

[7] Golsorkhi Mohammad S., Lu Dylan Dah-Chuan (2016). A decentralized control method for islanded microgrids under unbalanced conditions. IEEE Trans. Power Deliv..

[8] Shafiee Qobad, Guerrero Josep M., Vasquez Juan C. (2014). Distributed secondary control for islanded microgrids—a novel approach. IEEE Trans. Power Electron..

[9] Hug Gabriela, Kar Soummya, Wu Chenye (2015). Consensus+ innovations approach for distributed multiagent coordination in a microgrid. IEEE Trans. Smart Grid.

[10] Bani-Ahmed A., Rashidi M., Nasiri A., Hosseini H. (2018). Reliability analysis of a decentralized microgrid control architecture. IEEE Trans. Smart Grid.

[11] Caire R., Retiere N., Morin E., Fontela M., Hadjsaid N. (2003). Voltage management of distributed generation in distribution networks. IEEE Power Eng. Soc. General Meeting.

[12] Lee S.J. (2003). Calculation of optimal generation for system loss minimization using loss sensitivities derived by angle reference transposition. IEEE Trans. Power Syst..

[13] Khatod Dheeraj Kumar, Pant Vinay, Sharma Jaydev (2006). A novel approach for sensitivity calculations in the radial distribution system. IEEE Trans. Power Deliv..

[14] Lu X., Lai J., Yu X., Wang Y., Guerrero J.M. (Sept. 2018). Distributed coordination of islanded microgrid clusters using a two-layer intermittent communication network. IEEE Trans. Ind. Inf..

[15] Lai J., Zhou H., Lu X., Yu X., Hu W. (July 2016). Droop-based distributed cooperative control for microgrids with time-varying delays. IEEE Trans. Smart Grid.

[16] Lu X., Yu X., Lai J., Wang Y., Guerrero J.M. (July 2018). A novel distributed secondary coordination control approach for islanded microgrids. IEEE Trans. Smart Grid.

[17] Doost Mohammadi F., Keshtkar Vanashi H., Feliachi A. (March 2018). State-Space modeling, analysis, and distributed secondary frequency control of isolated microgrids. IEEE Trans. Energy Convers..

[18] Dehghan Banadaki A., Mohammadi F.D., Feliachi A. (2017). State space modeling of inverter based microgrids considering distributed secondary voltage control. North American Power Symposium (NAPS), Morgantown, WV, 2017.

[19] Olfati-Saber Reza, Fax J Alex, Murray Richard M (Jan. 2007). Consensus and cooperation in networked multi-agent systems. Proc.IEEE.

